# Manual Training

**Published:** 1893-02

**Authors:** S. B. Palmer

**Affiliations:** Syracuse, N. Y.


### MANUAL TRAINING.

BY S. B. PALMER, M.D.S., SYRACUSE, N. Y.

The addition of the third year to a course in dental colleges was a step in the right direction for progress in dental education.

With one-third more time for instruction, and to answer the demand for higher skill in the working of metals and in the construction of crowns and bridges, a course of study could be arranged to meet conditions at present not provided for.

There is not sufficient time spent with students in manual training to enable them to meet the requirements of practice. It is true this education may be acquired by a special course, at an expense which few can afford. Mechanical training comes in at the close of study rather than in the early part of the first term, so that the student is not able to enjoy its benefits throughout the full course.

I know from experience with the teaching of students early in my practice that a systematic course of study can be so arranged that manual training can be combined with the other studies and become pastime or recreation rather than a task. In training the fingers, work must be performed and directed in a way to produce something useful for the worker, and when this is done the mind becomes interested, the real labor forgotten.

I will venture to offer a few suggestions how it is possible to make mechanical training a recreation, and will simply mention what was laid out for students in the office thirty-five years ago.

Attention was first called to the furnishings and fixtures in the laboratory, then to the tools, and the advice given, "You will need all these, so commence and make for yourself what you can, and add as you may learn more, and make other things, until you have done all that the tools will enable you to do." Before the time of pupilage was over nearly every one had a complete outfit, with perhaps the additional assistance of a machinist to construct lathes, etc. The work was not limited to the laboratory. Dr. Hodson, of New York, is proud of a set of ivory-handled socket pluggers which are now in use by him, made during his office study in Syracuse.

For over twenty-five years I have not taken students, believing it best that young men intending to enter the profession should go at once to college rather than waste time in office service. In theory I was right; observation convinces me that practically I was wrong. The office student after graduating is much better prepared to enter practice than one who graduates without previous training.

It is not the professional teaching received in an office that places him in advance; many things must be unlearned, or still worse are retained; but the manual training received, which a private laboratory offers, is not enjoyed individually when a large class is being taught. In proof that mechanical training is of importance, though it be not directly in the line of college instruction, I will cite two cases. Two young men, by my advice, entered dental college without office study; both were limited in means and alike ambitious to succeed. After graduating in the two years' course, one came to me with a cast, for advice in a case of regulating. The case called for a simple gold appliance that required soldering in its construction. The young man said, "I have my diploma, but it is all a farce. I know nothing practically about soldering or working gold, and have it yet to learn." The other young man was proud to inform me at the close of his first term that he had added to his means by making, or teaching the boys how to make, metal cases. Where was the difference? The latter was a son of a fine mechanic, a model-maker, whose shop was a part of his dwelling, the boy's play-house; the use of tools was familiar to him, and once shown a thing to be made, and told how to make it, he could duplicate it. When a practical jeweller takes up dentistry he asks no one to assist him in constructing deposit cases.

Of course there will need to be an outlay on the part of the college to furnish facilities for this training, but there is demand for it, and there will be a supply. If colleges cannot do it individually, they could unite upon a plan to recommend the first year's course at a manual training college, and receive the diploma from that institution as one year on the course, and thus require but two terms. The course of study should be after that of the Chicago training school, or that of some universities, in the course of civil and electrical engineering, where each student has practical instructions in blacksmithing, wood-working, moulding, casting, the use of engine lathes, etc. The dental student should have for his lessons the making of things required in practice after graduating; he should make patterns for flasks for vulcanizing, turn out handles for bench tools, cut strips of brass, bend, solder, and turn up ferrules to fit the handles, and, later on, when chemistry is reached, nickel-plate his brass productions. He should cast flasks, forge bolts, and fit up the same; he should be taught to make and temper excavators and other fine instruments. This is not recommended for the saving of money, as no operator can afford to make fine tools, but as a part of educational training to meet emergencies that often occur in practice.

The above suggestions are based upon practical knowledge that students can be taught to work out their own manual training and regard it as pastime and not a task. What is true of private teaching can be enlarged upon and be made equally practical in college training.

